# What influences women’s decisions to participate in trials for prevention of venous thromboembolism during pregnancy and the puerperium: a qualitative study

**DOI:** 10.1186/s12884-025-07759-x

**Published:** 2025-06-04

**Authors:** Fiona C Sampson, Sarah Davis, Maxine Kuczawski, Rosemary Carser, Beverley J Hunt, Steve Goodacre, Abdullah Pandor, Catherine Nelson-Piercy, Jahnavi Daru

**Affiliations:** 1 Sheffield Centre for Health and Related Research (SCHARR), University of Sheffield, 30 Regent Street, Sheffield, S1 4DA UK; 2 PPI contributor,Thrombosis UK, PO Box 58, Llanwrda, UK; 3 Kings Healthcare Partners, Guy’s and St Thomas’ NHS Foundation Trust, London, UK; 4 Obstetric Medicine Department, Guy’s and St Thomas’ NHS Foundation Trust, London, UK; 5 Institute of Population Health Sciences, Queen Mary University of London, London, UK

**Keywords:** Trial participation, Qualitative research, Women’s health, Communication, Thromboprophylaxis, Pregnancy

## Abstract

**Background:**

Thromboprophlyaxis for the prevention of venous thromboembolism during pregnancy and the puerperium is widespread, but there is a lack of evidence on the risks and benefits of thromboprophylaxis within this population. Trials involving pregnant women often struggle to recruit and retain participants which makes It difficult to improve the evidence base. We undertook qualitative evaluation of patient perspectives of pregnancy/postpartum thromboprophylaxis to understand willingness to participate in future trials.

**Methods:**

We undertook four focus groups of women who had thromboprophylaxis due to prior VTE (*n* = 10) or been offered thromboprophylaxis due to other risk factors (*n* = 12) during pregnancy and the puerperium. Focus groups were held online between November 2021 and January 2022. We recruited via social media and national special interest groups representing diverse cultural and socio-economic backgrounds, sampling purposively for condition, age, ethnicity, and socio-economic status. Participants received a £50 voucher. We transcribed focus groups and analysed data using thematic analysis.

**Results:**

A lack of knowledge around the risks and benefits of thromboprophylaxis influenced how women perceived future trial participation. Limited understanding of thromboprophylaxis risks led to a lack of equipoise among participants who only identified benefits from treatment. Some women were unaware of why they had been given thromboprophylaxis but still perceived placebo as an inferior option. Concerns around injecting thromboprophylaxis were often minimised and ignored by healthcare professionals yet influenced treatment adherence. However, these negative experiences also motivated women to participate in future trials to receive a higher standard of care, as well as improving future care for others.

**Conclusions:**

Trial treatment adherence may be affected by negative experiences of injecting and limited understanding of why they had been offered thromboprophylaxis. To improve recruitment and retention in pregnancy and puerperium clinical trials, women need to be given clear explanations of the risks and benefits of treatment and understand where there is genuine clinical equipoise. Improved communication may also improve the experience and treatment adherence for women currently being offered thromboprophylaxis.

**Supplementary Information:**

The online version contains supplementary material available at 10.1186/s12884-025-07759-x.

## Introduction

Venous thromboembolism (VTE) is a leading cause of potentially avoidable direct maternal death [1]. Although incidence is low at a rate of 1–2 per 1000 deliveries, women are between four to five times more likely to develop deep vein thrombosis (DVT) and pulmonary embolism (PE) when pregnant and VTE remains a primary cause of maternal death worldwide [2–5]. Risk of VTE in pregnancy and the puerperium may be increasing due to changes in the health profile of women who become pregnant, due to increasing maternal age, increase in obesity rates and co-morbidities [6, 7]. Thromboprophylaxis with low molecular weight heparin (LMWH) has been shown to reduce VTE risk in medical and surgical patients but is associated with a slightly increased risk of bleeding [8].

Prescription of LMWH to prevent VTE is currently recommended for women considered to be at high risk of VTE during pregnancy or the puerperium [9, 10]. However, although thromboprophylaxis is widely accepted during pregnancy and the puerperium, high quality evidence on the risks and benefits of treatment within this population is lacking. The evidence about benefits and potential harms of offering thromboprophylaxis for prevention of VTE in women during pregnancy or the puerperium is highly uncertain, due limited high-quality trials that include pregnant women, particularly those without prior VTE [11], Risk assessment tools, which are used to assess suitability for thromboprophylaxis rely on trials from which pregnant women have been excluded, with evidence extrapolated from other populations or based on poor quality observational research [12].

In recognition of the need for an improved evidence base in this area, the UK NIHR Health Technology Assessment board wished to identify priority areas of research, recognising difficulties in recruitment of pregnant patients. To support a wider study using decision analysis modelling to understand where future studies should focus in order to reduce decision uncertainty in prescribing thromboprophylaxis during pregnancy and the puerperium, we undertook qualitative evaluation of stakeholder perspectives to understand issues affecting willingness to participate in future trials. We aimed to use women’s perspectives to understand what factors may influence women’s participation in trials of thromboprophylaxis during pregnancy and the puerperium.

## Methods

We used an inductive, naturalistic methodology to explore patient perceptions of participation in future trials. We undertook focus groups in order to elicit multiple views, generate data from group interaction and understand experiences and feelings that may be shared by different groups [13, 14].

### Recruitment

We organised four focus groups with women with experience of using low molecular weight heparin (LMWH), or who had been offered LMWH in pregnancy or the puerperium. We undertook 2 focus groups in each of the two populations: (1) women who have experienced DVT or PE during pregnancy or within six weeks after delivery and (2) women who have no prior VTE but have been offered thromboprophylaxis during pregnancy or within six weeks after delivery for other reasons. We aimed to recruit between four to eight participants to each group and selected four groups as our sample as a feasible recruitment target likely to achieve a high level of theme saturation [13].

We recruited participants via national special interest groups and relevant charities, selecting a number of special interest groups to help with recruitment who we considered to represent diverse cultural and socio-economic backgrounds in order to recruit as wide a range of participants as possible. Due to non-dominant ethnic groups being under-represented in health studies and in order to address the racial disparities in maternity outcomes [1, 15] we focussed particularly on reaching people from a range of ethnic backgrounds. We offered payment at a rate of double minimum wage, which has been suggested as a potential enabler to encouraging diversity of engagement and reported that we wanted to speak to people from ethnic minority groups within recruitment materials [15, 16]. We also advertised widely on social media using Twitter ™ (now X ™). We developed advertising and invitation materials with the PPI lead and explained that participants would receive a £50 shopping voucher following participation in the focus group.

### Sampling

We received 46 initial expressions of interest, with participants providing some brief description of their background within their emails (28 prior VTE, 18 no prior VTE). We emailed information sheets and consent forms to participants who expressed an interest and asked participants for details of whether they had prior VTE, age, sex, ethnicity and socio-economic status (highest educational award) (see supplementary file 1), using this data to achieve a purposive sample where possible. We did not receive participant details for all participants and included three respondents who did not complete the participant details but did complete the consent forms and provided us with details about their background (e.g. previous DVT). We received responses and completed information sheets from a total of 22 participants and arranged focus groups for those who responded via doodle polls.

### Focus groups

We undertook the focus groups using online Google Meets as were unable to undertake planned face-to-face focus groups due to Covid-19 restrictions. The focus groups took place between November 2021 and January 2022. The focus groups were led by a facilitator (FS) with a note-taker undertaking detailed notes. During the initial introduction, the facilitator summarised the purpose of the study, explained that the evidence base was poor and that trials to assess effectiveness of thromboprophylaxis had not included pregnant women. Focus group topic guides (see supplementary file 2) were designed as broad guides to enable participants to talk about what was important to them whilst ensuring that they addressed each of the broad questions relating to their experience of using LMWH, how risks and benefits of LMWH were communicated and their perspectives of participating in future trials of LMWH versus placebo. The facilitator summarised what participants reported at intervals during the focus groups to clarify understanding and allow correction of any misinterpretations. Focus groups were recorded and transcribed verbatim.

### Public and patient involvement (PPI)

The project team included a patient and public (PPI) representative (RC) from Thrombosis UK, who has relevant personal experience of VTE. She contributed to the design of the study, attended project management group meetings and was instrumental in developing questions for the focus groups and leading the recruitment strategy.

### Analysis

We analysed data using a thematic analysis approach according to the principles of Braun & Clarke [17]. Transcripts were read and checked by two researchers (FS, MK) who then independently identified initial codes. Following discussion of codes, the researchers then identified patterns of meaning (themes) and explored the relationships between the themes. Positionality of researchers was considered throughout; both researchers are white, female Health Service Researchers with experience of pregnancy and childbirth, positive and negative experiences of pregnancy-related care and childbirth, but not of being offered thromboprophylaxis. While our personal experiences provide empathy and understanding, they also limit our direct understanding of the specific challenges and concerns associated with thromboprophylaxis. To address this, we collaborated closely with a patient and public involvement (PPI) representative who has first-hand experience with VTE during pregnancy. This collaboration ensured that the research questions and approach were relevant and meaningful to the participants’ lived experiences.

### Findings

We undertook interviews with 22 participants over a total of four focus groups, two of which involved patients with prior VTE (*n* = 7, *n* = 3) and two of which involved patients who had been offered thromboprophylaxis but had no history of prior VTE (*n* = 6, *n* = 6). Details of participants are detailed below in Table 1.

**Table 1:** Details of focus group participants

Focus group	Participant ID	Age group	Ethnicity	Employment	Education	Previous DVT/PE
1	W1P1	25–34	White/Caucasian	Full time	Bachelor’s degree +	Both
1	W1P2	55+	White/Caucasian	Full time	Doctorate degree	PE
1	W1P3	25–34	White/Caucasian	Student	Bachelors degree	Both
1	W1P4	35–44	White/Caucasian	Student	Masters degree	Both
1	W1P5	25–34	White/Caucasian	Part time	Associate degree	Both
1	W1P6	35–44	White/Caucasian	Part time	High school	PE
1	W1P7	35–44	Asian/Asian British	Part time	Bachelors degree	PE
2	W2P1	25–34	White/Caucasian	Full time	Bachelors degree	PE
2	W2P2	35–44	Asian/Asian British	Full time	Bachelors degree +	DVT
2	W2P3	25–34	White/Caucasian	Part time	Bachelors degree	DVT
3	W3P1	35–44	White/Caucasian	Part time	High school	None
3	W3P2	N/A	N/A	N/A	N/A	None
3	W3P3	N/A	N/A	N/A	N/A	None
3	W3P4	35–44	White/Caucasian	Unemployed	Bachelors degree	None
3	W3P5	N/A	N/A	N/A	N/A	None
3	W3P6	45–54	White/Caucasian	Full time	Doctorate degree	None
4	W4P1	35–44	White/Caucasian	Part time	Masters degree	None
4	W4P2	35–44	White/Caucasian	Full time	Masters degree	None
4	W4P3	45–54	White/Caucasian	Full time	Doctorate degree	None
4	W4P4	35–44	White/Caucasian	Full time	Masters degree	None
4	W4P5	45–54	White/Caucasian	Part time	Bachelors degree	None
4	W4P6	35–44	White/Caucasian	Part time	Masters degree	None

## Results

We identified a unifying theme that a lack of awareness around the risks and benefits of thromboprophylaxis in VTE influenced how women perceived future trial participation. We explain this within the following four subthemes.

### Pregnant women receive limited information about VTE or risk and benefits of thromboprophylaxis during pregnancy or post-partum. Adherence with treatment may be affected by lack of understanding of the rationale behind treatment

Participants described receiving limited information about VTE or thromboprophylaxis in terms of either risk of VTE during pregnancy, understanding why they had been given anticoagulants (particularly for low-risk participants) or the risks and benefits of treatment. Some participants who had VTE during pregnancy were unaware that this was a risk of pregnancy and reported lack of information regarding their treatment.

*W2P3: No-one has the time to talk to you in pregnancy and I hardly had no more than 10 min with the midwife during pregnancy before the DVT. […] I didn’t even know blood clots could happen during pregnancy at that point.*

*W1P5: Everything was so quick I didn’t take anything in at all. The only risks and benefits that were given to me was with the consultant later around being induced and risks and benefits there*,* but nothing around going on the injections in the beginning.*

Most participants without prior VTE had some general awareness about DVT and PE but reported that the risks of VTE were not generally discussed as part of their maternity care and could recall little or no discussion of the increased risk of VTE during or after pregnancy. During the focus groups they described assumptions or guesses made about why they had been given thromboprophylaxis, based on their experiences of childbirth or knowledge of risk factors from other sources.

*W3P3: I’m not aware of the risks [of thromboprophylaxis]*,* so no one’s explained it to me*,* and I didn’t feel that there was much point searching for it after being on it for a while*,* so that was it.*

*W4P6: On reflection*,* I had lost a lot of blood during the birth*,* during the surgery so I was put two together myself there. However*,* I stopped after 10 days because just the stress of having to it with two little ones running round was just too much and I didn’t have any blood pressure issues.*

*W3P4 Hi*,* erm*,* so I was on the blood thinning injections for 10 days after giving birth*,* and I*,* to me it was because I lost*,* I lost 1.5 L of blood and I had pre-eclampsia*,* so they’re the reasons I believe that I was put on it*,* but I wasn’t actually told why I needed them*,* I was just discharged with them and I didn’t really question it.*

This lack of awareness of risk factors for VTE and/or rationale for treatment may have affected treatment adherence, with participants describing stopping treatment early because they did not understand why they should be taking it. Others reluctantly complied with treatment that they felt they were expected to take, but were unhappy at doing so.

*W2P2: The first time round I just took the injections because I was told to*,* but if I missed I wasn’t really bothered*.

*W3P4… I probably did about 7 injections in total*,* including the two in hospital. So I missed three basically.*

### Int: right. And did you understand why you were doing it?

*W3P4: No. I think that ‘s probably why I didn’t really continue.*

### Due to lack of Understanding of risks associated with thromboprophylaxis, participants perceived entering a trial to be withholding treatment due to the risk of not receiving thromboprophylaxis

Participants did not fully recognise the inadequate evidence base underpinning current guidelines and perceived prescription of thromboprophylaxis as potentially life-saving and best practice. They believed that they had been offered the best treatment by their clinicians and perceived the introduction of a trial as potentially withholding treatment (placebo) rather than offering a choice of treatments where the current evidence is unclear.

*W3P6: I’ve been really anxious. If it had been a choice of you know*,* “you’re not going to get them*,* but if we put you on a trial there is a fifty-fifty chance you’ll get them*,* or placebo”*,* then I’d have gone for it*,* but if it was a case of “you can have them or you can go into a trial”*,* I would definitely have wanted them*,* because of my anxiety around being in a you know*,* over coagulated state not having the anticoagulants.*

*I’m really struggling to see how you could give somebody nothing*,* if they had risk factors (W1P5)*.

*W4P3: I guess it’s because it would be a trial to remove something that’s commonly practiced*,* I’d need to know why is it*,* I suppose I’d just need to know more and have more questions.*

Participants with greater awareness of the impact of DVT and PE either from previous experience of VTE or knowing someone who had it appeared less willing to participate in future research. These patients (e.g. those on long-term thromboprophylaxis) reported having little agency and being unable to take part in trials as a placebo would not be a feasible option. Other participants who were unaware of why they had been given thromboprophylaxis still perceived receiving a placebo as an inferior option and entering a trial as a risk.

*W1P7: I couldn’t choose not to take the blood thinners.*

*W4P1: Of course I took that [thromboprophylaxis]*,* but if somebody mentioned the word ‘trial’ to me*,* I would have said no because I would not have put anything at risk for me or my children.*

### Listening to and Understanding women’s concerns May decrease likelihood of attrition from treatment group

Participants in all four focus groups spent a significant amount of time discussing the side-effects associated with self-injection, which they felt would prevent adherence to treatment for people who had not had previous experience of VTE. They described having their concerns minimised and ignored by healthcare professionals, particularly their concerns about side-effects of bruising and lumps associated with injecting and lack of understanding of what was ‘normal’. Although reportedly dismissed by healthcare professionals, these experiences were significant enough for some to avoid having further children.

*W2P2: I think my bruising hurt more than my C-section […] I certainly would never consider having another baby now because of the thought of injecting myself.*

*W1P2: I decided not to have a second baby because I couldn’t’ face the idea of taking those injections twice a day […] that was a really big issue for me and I really felt that this was minimised in the expectation that it will be fine*.

These poor care experiences motivated women to participate in future trials to receive a higher standard of monitoring and care, as well as providing an opportunity to improve future care for others. They described feeling safer as part of a trial due to increased monitoring and a higher standard of care.

*W3P3: I think for me it’s*,* it would be about clarity of information and actually*,* almost providing “okay this what we’re trying to research*,* but if you join this trial*,* we’ll give you xyz”*,* so you get extra check-ups*,* extra scans*,* extra*,* if you were doing it obviously pre-*,* during your pregnancy so that you were confident that regardless of whether you were or you weren’t*,* your standard of care is almost raised up another level*,* so you weren’t just being looked after*,* you were being like gold standard*,* you know you were getting check-ups*,* you know*,* once a month*.

*W2p1 I think for me it would be how closely you’re going to monitor it*,* you know*,* like what sort of tests I suppose*,* would you be doing to monitor if I’m getting a blood clot anywhere? You know*,* how closely are you going to be looking after me kind of thing? For me personally. I’d want to know “are you going to see me quite often?*

### Information about risk and benefits of VTE and potential recruitment into trials should be communicated during pregnancy rather than post-partum

Women described the vulnerability of their post-partum ‘headspace’ and did not feel that this would be an appropriate time for discussion or considering risks of treatment or being recruited into trials. Some participants without prior VTE were offered thromboprophylaxis after giving birth, having had no prior discussions about the possibility of this happening. They described how they accepted whatever the doctor or midwife was offering without question at the time and a need to trust what was being offered. They expressed the need for trial recruitment particularly to happen during pregnancy when they had improved capacity to consider information and consent.

*W4P1: I think if I was given the information at like*,* I don’t know*,* an 18 week or 20 week appointment around ‘actually this is what could happen and this is what we might need to give you’ I feel like I would have been in a much better place and even had the headspace to actually […] sit down and actually thoroughly be able to formulate questions.*

*W4P6:…like I say*,* high quality of information with a trusted person during pregnancy so you had the chance to have some form of better clarity of decision making*,* and then the opportunity to probably opt-out just depending on kind of how the birth went*,* how it felt.*

## Discussion

We identified that there was a lack of patient information around the risks and benefits of thromboprophylaxis in VTE during pregnancy and the puerperium. This influenced how women perceived future trial participation. Due to lack of understanding of risks associated with thromboprophylaxis, participants perceived entering a trial could result in them receiving placebo which they perceived as having treatment withheld, having previously assumed their clinician had provided an effective treatment. Patients with VTE risk factors other than previous VTE, were often unaware of why they had been given thromboprophylaxis but still perceived receiving a placebo as an inferior option. Both groups reported lack of information about the risks and benefits of pharmacological thromboprophylaxis.

Women described having their concerns minimised and ignored by healthcare professionals, particularly their concerns about side-effects of injecting. These poor care experiences motivated women to participate in future trials to receive a higher standard of monitoring and care, as well as providing an opportunity to improve future care for others. Adherence to treatment may be affected by negative experiences of injecting and limited understanding of reasons why they had been offered thromboprophylaxis. Gee et al. similarly identified lack of information throughout their care and lack of awareness of VTE prior to diagnosis in a phenomenological study of 9 patients with pregnancy-related venous thrombosis [18]. 

Other studies have focused on barriers and enablers to trial participation within women who were pregnant and post-partum and identified similar barriers. Van der Zande et al. (2018) undertook a systematic review of women’s reasons for participating in research and identified a reluctance to take placebos as part of an RCT and disbelief in equipoise, wanting reassurance that they would receive the intervention [19]. This suggests that the limited understanding of evidence-base and risk/benefit profiles of treatments applies to wider contexts. They similarly identified that indirect benefits, including additional monitoring or receiving better treatment were incentives to taking part in trials, particularly for more invasive interventions, which reflects our findings that women were motivated to participate in future trials to receive a higher standard of care. They also highlighted discomfort due to tests such as needle pricks as being a barrier to participation.

Stromner et al. identified that women who were likely to take part in clinical trials during pregnancy had prior knowledge of the importance of the study topic, with women who declined participation having limited previous knowledge [20]. This supports our finding that limited knowledge about the risks and benefits of VTE thromboprophylaxis may limit adherence or participation in trials.

Other studies have explored likelihood of using thromboprophylaxis during pregnancy for women with prior VTE. Patel et al. used a structured questionnaire to assess adherence to enoxaparin during pregnancy and identified high rates of adherence amongst women with prior VTE [21]. Bates et al. and Eckman et al. used structured interviews and direct choice exercises to understand willingness to accept use of LMWH during pregnancy for different risk thresholds, amongst women with history of VTE who were pregnant or planning pregnancy [22, 23]. Bates et al. and Eckman et al. both identified a high degree of variation in response, with Eckman et al. identifying discordance between the direct-choice of the women and the risk identified by the model. Bates et al. identified that high risk patients were more likely to take LMWH than lower risk patients, but did not explore reasons for this difference. These studies suggest that adherence may be higher for patients with prior experience of VTE but also suggests that other factors are involved. None of the studies used qualitative methods to explore these reasons further.

Smyth identified that pregnant women were more likely to take part in trials when they had high levels of trust in clinicians, suggesting that clinicians have a key role to play in providing appropriate information and influencing women’s decisions to take part [24]. Hanrahan et al. reported a ‘recruiter knows best’ attitude of healthcare professionals in recruiting pregnant women to trials, and paternalistic language suggesting a power imbalance between healthcare professionals and pregnant women. Indeed, much of the qualitative evidence of barriers to trial recruitment currently focuses on healthcare professional views of barriers to recruitment [24–28]. Improved provision of evidence-based information on the risks and benefits of treatment may help to address this imbalance of power.

### Strengths and limitations

These focus groups asked women with previous, often recent, experience of being offered thromboprophylaxis during pregnancy for their views of trial recruitment in an area where they had experience. The focus groups offered opportunities for interaction which enabled participants to focus on areas that were important to them (e.g. side-effects of injecting) which helped to reveal important aspects of patient experience that were previously under-estimated or disregarded. However, this focus did mean that less time was dedicated to exploring individual perspectives about involvement in clinical trials than may have been possible had we used semi-structured interviews.

Although we aimed to recruit a diverse group of participants, the participants recruited were predominately white and highly educated, which reduces the transferability of findings to other groups. We did not specify a maximum amount of time since pregnancy which may have led to some recall bias. Recruitment via user groups may mean our sample had a higher level of health literacy and engagement with research than the general population. Poor care experiences may have led to a biased sample of women taking part in the research. However, there is a wealth of evidence demonstrating that poor care experiences in maternity care are widespread which suggests that our sample were not highly unrepresentative [29–31]. Despite attempts to recruit from non-dominant ethnic groups, none of our respondents who provided demographic details identified themselves as black or mixed/multiple ethnic groups, two identified as Asian/Asian British and 20 as White/Caucasian. Due to the Covid-19 pandemic our recruitment approaches were impersonal (i.e. social media, email), focus groups were conducted in English and materials were not made available in other languages which may have been potential barriers for recruitment from non-dominant ethnic groups [16]. However, given that improved communication has been highlighted as key to improving trial recruitment, it is likely that the biased sample may under-represent some of the concerns that would be held within a more diverse population. The UK Parliamentary and Health Service Ombudsman report identified that women were not being listened to when raising concerns in pregnancy, and that this was worse for women from Asian, Black or Mixed ethnic backgrounds [29]. 

Most participants had recent experience of childbirth and/or thromboprophylaxis and were able to provide their theoretical perspective of what would be important to them when being recruited to a trial. Inclusion of nulliparous women or those with no prior experience of being offered thromboprophylaxis would have enabled a more naïve stance that reflects likely future trial participants. However, perspectives of nulliparous women may have over-estimated likelihood of trial engagement due to lack of awareness of the realities of childbirth or post-partum experiences.

Although we aimed to focus on future trial participation, women focussed on their past experiences which shaped their motivations to be involved in future trials. However, future trials are likely to also recruit nulliparous participants whose lack of prior experience of thromboprophylaxis may be more open to understanding equipoise, particularly for those without prior VTE. Although we asked for participants who had been offered thromboprophylaxis, we had few respondents who had been offered but refused thromboprophylaxis, which limits transferability of findings to this group.

### Implications

Pregnant women receive limited information about VTE or risks and benefits of thromboprophylaxis during pregnancy or post-partum and those without prior VTE often do not understand why they have been given the treatment. Clearer information about the risks and benefits of thromboprophylaxis and an increased understanding of the rationale behind treatment may improve treatment adherence and recruitment and adherence to trials. Improved communication may also improve the experience and treatment adherence for women currently being offered thromboprophylaxis.

Negative experiences associated with injections were important to the women in this study yet minimised by healthcare professionals. In order to maximise participation in trials and reduce attrition these concerns need to recognised and addressed, not dismissed. Provision of honest information about potential side-effects of injections, an understanding of what is ‘normal’ as well as advice about how to decrease potential side-effects (e.g. use of ice) may help women make informed choices about taking part in clinical trials, and complying with treatment.

Discussion of VTE during pregnancy as well as consideration of why thromboprophylaxis may be required is needed during pregnancy when women are more able to consider treatment options rationally, and discuss with partners, family and friends.

## Conclusions

In order to improve the recruitment and retention of women in pregnancy and puerperium into clinical trials, women need to be given clear explanations of the risks and benefits of the treatment and understand where there is genuine clinical equipoise. This requires clear communication of the research evidence underpinning treatment options.

## Electronic supplementary material

Below is the link to the electronic supplementary material.

Supplementary Material 1

Supplementary Material 2

Supplementary Material 3

## Data Availability

Data will not be made publicly available to protect the participant’s identity. Permission to share anonymised quotations only was granted by participants. Permission to share original data was not granted by the participants. For further information about data please contact f.c.sampson@sheffield.ac.uk.

## Declarations

### Copyright/license for publication

All work funded by the NIHR is subject to Crown Copyright. We are therefore unable to assign copyright to the journal but will grant a non-exclusive licence (CC-BY) on a worldwide basis to permit this article (if accepted) to be published in BMC Pregnancy and Childcare.
