# Supplementary material for: What influences women’s decisions to participate in trials for prevention of venous thromboembolism during pregnancy and the puerperium: a qualitative study
Source: BMC Pregnancy Childbirth. 2025 Jun 4;25:651. doi: 10.1186/s12884-025-07759-x (PMC12135286; doi:10.1186/s12884-025-07759-x)
Supplement: Supplementary file 1 — Supplementary Material 1 [file 12884_2025_7759_MOESM1_ESM.docx]

Q1)   Please state your age group:

1. Below 18
2. 18-24
3. 35-44
4. 45-54
5. 55+

Q2)   What is your ethnic background? Choose from one option that best describes your ethnic group or background

1. Asian/Asian British
2. Black / African / Caribbean / Black British
3. Mixed / Multiple ethnic groups
4. White / Caucasian
5. Other ethnic group
6. Prefer not to say

Q3: Which of the following best describes your current employment status?

a)       Full-time employment

b)      Self-employed

c)       Part-time employment

d)      Underemployed (wage is below industry average)

e)      Full time freelancing

f)        Unemployed (looking for work)

g)       Unemployed (not looking for work)

h)      Student

i)        Inability to work

j)        Other

Q4: Please select the highest level of education that you have attained?

a)       Doctorate degree

b)      Master’s degree

c)       Bachelor’s degree

d)      Associate degree

e)      Trade/technical/vocational training

f)        High school/college graduate, diploma or equivalent

g)       Some high school

h)      Other

i)        Prefer not to say
