# Supplementary material for: What influences women’s decisions to participate in trials for prevention of venous thromboembolism during pregnancy and the puerperium: a qualitative study
Source: BMC Pregnancy Childbirth. 2025 Jun 4;25:651. doi: 10.1186/s12884-025-07759-x (PMC12135286; doi:10.1186/s12884-025-07759-x)
Supplement: Supplementary file 2 — Supplementary Material 2 [file 12884_2025_7759_MOESM2_ESM.docx]

**Suggested script for introduction**

Thanks for coming today. (Talk about the workshop and how it will all work. Going to audio record. Request no-one takes photos or video. Need for confidentiality.). If you need to dip in and out, that’s fine. If feel distressed or want to back out at any stage, please do so, don’t need to give an explanation.

(Talk about payment). Going to explain how this workshop will happen. I’m going to give you a brief introduction to the project, then I’m going to ask for your thoughts on various things. There are no right and wrong answers – just want your opinions. Covid – although everything we’ve done recently has been dominated by covid, would like to avoid talking about it where possible.

Will have a break for 5 mins after an hour.

You have all responded as you have previous experience of blood clots and have had to take blood thinners during pregnancy / have been offered blood thinners during pregnancy. Despite there being national guidance about who should receive blood thinners, the number of people presenting with blood clots during and shortly after pregnancy has not changed much over the years and we are doing a research project to understand where there is a need for clearer evidence. Current guidelines recommend giving blood thinners based on different risk factors, which can include previous clots, pre-existing clotting disorders, body mass index, age etc. These recommendations have been based on the results of different clinical trials that have shown how effective blood thinners are for people with different risk factors. However, most of the clinical trials on which these recommendations were based did not include pregnant women, and studies that did include pregnant women struggled to recruit enough people to get meaningful results. Our research study so far has identified areas where it would be most useful to have further evidence from clinical trials to understand how effective blood thinners are people with different risk factors.

We did a systematic review of the existing research literature to find all existing evidence for how effective treatments are for preventing further clots in pregnancy, or after giving birth and have undertaken mathematical modelling to understand which areas have the highest levels of uncertainty and would benefit most from evidence from randomised controlled trials. Before we report this to the funders, we want to understand a bit about whether trials would actually be feasible, and whether pregnant women would be willing to take part in trials. We want to speak to you as you have real-world experience of having been offered blood thinners to help us to understand what people might think if asked to take part in research in future. (For 1^st^ groups: As you have previously had DVT, your risks and perceptions of risks may be different.)

**Questions (cover in any order – adapt for previous DVT group)**

To start off with, can you give us a bit of background and tell me a bit about how you were told you would need blood thinners (prompt - how potential risks and benefits were explained)

Can you tell me a bit about your experience of taking blood thinners (prompt – did you take them as prescribed? Practical issues).

When you were asked to take heparin, did the doctor or midwife explain why you needed it?

Next, can we talk about how you think you might respond if a doctor or nurse explained that the evidence for blood thinners in your particular group was not very clear, and that they would like you to take part in a randomised controlled trial where you would be randomly allocated to either receive blood thinners or no treatment.

If instead of being told you needed to take heparin, you were told that there was not yet enough evidence about whether heparin was needed for your population, do you think you would have been willing to take part in a trial?

Would you be willing to take part in a trial?

Is there any further information that might help you make the decision whether to take part in a trial? (Prompt – concerns about taking part, potential benefits to being in a study, what might make you more willing to take part?)

When do you think would be the best time to make these decisions (prompt - during pregnancy / shortly after giving birth?

How would you feel about going through pregnancy without taking blood thinners when randomised to a trial?

Some types of trial will involve some hospitals giving blood thinners to a group of patients, and others not, rather than some individuals being given blood thinners. How do you feel about this?

Would you prefer the hospital to be randomised, or the individual? What would influence your decision? Who would influence your decision?

Some types of trial will involve some hospitals giving blood thinners to a group of patients, and others not, rather than some individuals being given blood thinners. How do you feel about this?

(Randomised controlled trials will only be allowed for treatments where there is what is known as ‘clinical equipoise’. This means that there is no clear evidence of which treatment is better. Ethical approval for a trial will only be allowed where this is the case. Sometimes, research uses ‘cluster randomised controlled trials’. This is where the treatments are allocated according to, for example, the hospital that is providing the treatment, or the doctor that is providing treatment, rather than individuals. This may mean that one hospital may offer heparin as part of a study and another may not. )

At 2 hours – end discussion. Thank all for attending and remind them about the process for receiving payment.
